# Experimental drought reduces the transfer of recently fixed plant carbon to soil microbes and alters the bacterial community composition in a mountain meadow

**DOI:** 10.1111/nph.12569

**Published:** 2013-10-31

**Authors:** Lucia Fuchslueger, Michael Bahn, Karina Fritz, Roland Hasibeder, Andreas Richter

**Affiliations:** 1 Department of Microbiology and Ecosystem Science, University of ViennaAlthanstr. 14, A-1090, Vienna, Austria; 2 Institute of Ecology, University of InnsbruckInnsbruck, Austria

**Keywords:** ^13^C pulse-labelling, carbon allocation, drought, microbial community composition, mountain grassland, mowing, phospholipid fatty acids

## Abstract

- Drought affects plants and soil microorganisms, but it is still not clear how it alters the carbon (C) transfer at the plant–microbial interface. Here, we tested direct and indirect effects of drought on soil microbes and microbial turnover of recent plant-derived C in a mountain meadow.
- Microbial community composition was assessed using phospholipid fatty acids (PLFAs); the allocation of recent plant-derived C to microbial groups was analysed by pulse-labelling of canopy sections with ^13^CO_2_ and the subsequent tracing of the label into microbial PLFAs.
- Microbial biomass was significantly higher in plots exposed to a severe experimental drought. In addition, drought induced a shift of the microbial community composition, mainly driven by an increase of Gram-positive bacteria. Drought reduced belowground C allocation, but not the transfer of recently plant-assimilated C to fungi, and in particular reduced tracer uptake by bacteria. This was accompanied by an increase of ^13^C in the extractable organic C pool during drought, which was even more pronounced after plots were mown.
- We conclude that drought weakened the link between plant and bacterial, but not fungal, C turnover, and facilitated the growth of potentially slow-growing, drought-adapted soil microbes, such as Gram-positive bacteria.

## Introduction

Drought has the potential to lead to severe imbalances in the terrestrial carbon (C) cycle, by affecting the production of organic matter by plants and decomposition by microorganisms (Ciais *et al*., 2005; Schwalm *et al*., 2010; Reichstein *et al*., 2013). Drought directly alters the physical environment for soil microorganisms and plants: it decreases soil water content and concomitantly increases the proportion of oxygen-filled soil pores (Schimel *et al*., 2007; Manzoni *et al*., 2012a; Moyano *et al*., 2013); it reduces the mobility of nutrients in the soil, thereby disconnecting organisms from substrates, while nutrient concentrations in the remaining soil water increase (Schjønning *et al*., 2003; Schimel *et al*., 2007). Thus, drought has a strong but equivocal effect on nutrient availability.

The response of a microbial community to drought depends on the physiological tolerance and metabolic flexibility of the constituent microbes (Allison & Martiny, 2008). Generally, fungi are thought to be more tolerant to dry periods than bacteria (Schimel *et al*., 2007; Strickland & Rousk, 2010; Manzoni *et al*., 2012a). They are able to create large hyphal networks that facilitate nutrient and water transfer over long distances, and to explore water-filled soil pores not accessible for plant roots (Allen, 2007; Joergensen & Wichern, 2008), and they have lower nutrient requirements than bacteria (Strickland & Rousk, 2010). Mycorrhizal fungi are directly connected to plant roots from which they obtain recently assimilated C (10–30% of the net primary production; Allen, 2007; Jones *et al*., 2009; de Deyn *et al*., 2011). Mycorrhizas can even enhance water supply for plants during drought by taking up water from smaller soil pores (Wardle *et al*., 2004; Allen, 2007). Soil ecosystems dominated by fungi may therefore be considered to be less sensitive to drought than bacteria-dominated soils (Yuste *et al*., 2011; de Vries *et al*., 2012).

Bacteria, however, are expected to inhabit smaller soil pores, and may be protected for longer from desiccation (Moyano *et al*., 2013); nonetheless, they have to balance the increasing osmotic potential of the soil solution (Schimel *et al*., 2007). Alternatively, they can endure unfavourable conditions by shifting to dormancy or producing cysts (Schimel *et al*., 2007; Lennon & Jones, 2011). Gram-positive bacteria appear to be more resistant to drought than Gram-negative bacteria, because of their thicker peptidoglycan cell wall layer (Schimel *et al*., 2007; Manzoni *et al*., 2012a). Genera of Gram-positive bacteria (e.g. *Firmicutes*) have been termed ‘drought-adapted generalists’ (Lennon *et al*., 2012). Overall, drought may select for more resistant microbial groups, which can result in the shift of an existing microbial community (Castro *et al*., 2010), and affect microbial-driven ecosystem functions by changing their activities (Schimel *et al*., 2007; Allison & Martiny, 2008; Lennon & Jones, 2011; Wallenstein & Hall, 2012).

In addition to direct physical effects, drought can affect soil microbes by altering the input of plant C into the rhizosphere (Bardgett *et al*., 2008). On the one hand, drought may alter fine-root turnover (Chaves *et al*., 2003), and on the other hand drought may affect the input of root exudates (including secretions, lysates from border cells and mucilage; see Jones *et al*., 2009). Root exudates represent an important source of organic C for soil microorganisms in the rhizosphere and enhance soil organic matter decomposition (i.e. the ‘priming effect’; e.g. Fontaine *et al*., 2003; Kuzyakov, 2010), which in turn makes nutrients accessible for microbial as well as for plant uptake. During drought periods, plants may alter belowground C allocation (Chaves *et al*., 2003; Ruehr *et al*., 2009; Albert *et al*., 2011; McDowell, 2011; Manzoni *et al*., 2012b). This may severely affect the quantity and quality of C available for soil microbes in the rhizosphere, such as fungi and Gram-negative bacteria, which seem to be tightly connected to recently assimilated plant C (Denef *et al*., 2009; de Deyn *et al*., 2011; Bahn *et al*., 2013). Nevertheless, it is not clear how drought affects processes at the root–soil interface (Bardgett *et al*., 2008; Compant *et al*., 2010; Sanaullah *et al*., 2011). Moreover, it is poorly understood how drought affects belowground C allocation in usually well-watered ecosystems, such as grasslands in the European Alps (Wieser *et al*., 2008), which are predicted to experience more frequent drought periods in the near future (Schär *et al*., 2004; IPCC, 2007; Seneviratne *et al*., 2010).

In many European grasslands, mowing, that is, clipping and subsequent harvesting of aboveground plant biomass, is a common management practice and shapes plant and microbial communities, as well as nutrient composition in the soil (Bardgett *et al*., 2001; Klumpp *et al*., 2011; de Vries *et al*., 2012; Meyer *et al*., 2012; Shahzad *et al*., 2012). In contrast to drought, mowing is an immediate disturbance, abruptly changing the soil microclimate (Bahn *et al*., 2006) and C input, as plant roots release a pulse of low-molecular-weight compounds (Paterson & Sim, 1999; Hamilton *et al*., 2008; Henry *et al*., 2008). This has been shown to induce a transient increase in microbial nitrogen (N) mineralization, allowing the higher N demand of plants to be met to rebuild biomass (Paterson & Sim, 1999; Hamilton *et al*., 2008; Henry *et al*., 2008; Cheng *et al*., 2011; Shahzad *et al*., 2012). It is, however, still unexplored whether drought alters such effects of mowing, but it may be speculated that plants under drought conditions may have lower nonstructural C reserves, potentially decreasing the intensity of the C pulse.

In this study, we therefore aimed to assess the direct and indirect plant-mediated effects of a severe summer drought on microbial processes and community composition in a mountain meadow. We asked the questions of how drought under field conditions affects the biomass of plants and the abundance of microbial groups, how it affects the transfer of C from plants to microbes, and how it alters short-term C turnover in grasslands after mowing. Specifically, we hypothesized that drought decreases plant C pools, thereby reducing the availability of recently fixed plant C for microbes in the rhizosphere; and that, overall, drought decreases microbial biomass, reducing the abundance of bacteria more strongly than that of fungi. Finally, we hypothesized that drought reduces the pulse of recently assimilated C to the soil after mowing compared with controls. We experimentally simulated an extended drought period in a mountain meadow in the Austrian Central Alps. Soil microbial biomass and community composition were determined using phospholipid fatty acids (PLFAs). To investigate the allocation of recently assimilated C to microbial biomass, we pulse-labelled plants with ^13^CO_2_ and traced labelled C from plants via the extractable organic C (EOC) pool to microbial PLFAs. Both controls and drought plots were mown towards the end of experimental drought and after labelling.

## Materials and Methods

### Study site

The study area was located in the Austrian Central Alps near Neustift, Stubai Valley (47°07′45″N, 11°18′20″E); the sampling site, a mountain meadow, was situated at 1850 m above sea level. Mean annual temperature was *c*. 3.0°C; mean annual precipitation was *c*. 1097 mm. Soils were classified as dystric cambisols (Food and Agriculture Organization of the United Nations, soil classification system) with a pH (in CaCl_2_) of 4.9 (Meyer *et al*., 2012). The study site is characterized by high plant productivity, with peak above- and belowground biomasses ranging between 240 and 440  g m^−2^ and between 420 and 980 g m^−2^, respectively (Bahn *et al*., 2006; Schmitt *et al*., 2010), as well as comparatively high soil respiration rates (Bahn *et al*., 2010). The experimental site was part of a meadow that is briefly grazed by cattle in spring and autumn, and cut for haying once a year at the end of July. The vegetation is dominated by perennial grasses and herbs including *Anthoxanthum odoratum* L., *Festuca rubra* L., *Alchemilla vulgaris* L., *Leontodon hispidus* L. and *Trifolium repens* L. (Bahn *et al*., 2009; Schmitt *et al*., 2010).

### Experimental set-up

**Figure 1:**
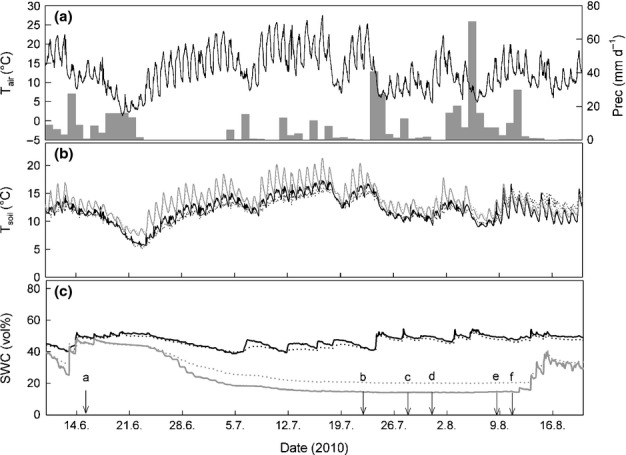
Microclimate during the drought experiment: (a) air temperature (*T*_air_; in °C 0.5 h^−1^) and precipitation (prec; in mm d^−1^); (b) soil temperature (*T*_soil_; in °C 0.5 h^−1^) and (c) volumetric soil water content (SWC; in %) at 5 (solid lines) and 10 cm soil depth (dotted lines) in control (black) and drought (grey) plots. Arrows with lowercase letters in (c) indicate dates of action: ‘a’ marks the beginning of the drought treatment; ‘b’, ‘c’ and ‘d’ indicate dates of pulse-labelling; ‘e’ indicates mowing and ‘f’ indicates rewetting and thus the end of the drought treatment.

We simulated an extended summer drought event by installing rain-out shelters (3.0 × 3.5 m; *n *=* *3) with light- and UV-B-permeable plastic foil (UV B Window; Folitec GmbH, Westerburg, Germany; light permeability *c*. 95%; UV-B permeability > 70%) to exclude any precipitation. In the centre of each shelter as well as in nearby established controls, plastic frames (1 × 1 m) were carefully placed into the upper 3 cm of soil. Rain-out shelters were left on the plots for 8 wk (16 June 2010 to 11 August 2010; Fig. 1); to detect the effect of mowing and to maintain the usual land-management practice in this meadow (Schmitt *et al*., 2010), the aboveground biomass of the complete site was cut and removed before the end of the drought treatment (8 and 9 August 2010). After plots had been mown (11 August 2010), they received 20 l m^−2^ of previously collected rainwater (= 20 mm precipitation) over a time period of 3 min, mimicking a heavy precipitation event, and subsequently rain-out shelters were removed. Precipitation, air temperature (*T*_air_) and soil temperature (*T*_soil_) (S-TMB-M006 with HOBO Micro Station Data Logger H21-002; Onset Computer Corp., Bourne, MA, USA) and soil water content (SWC; ECH2O EC-5 with data Logger EM50; Decagon Devices Inc., Pullman, WA, USA) at 5 and 10 cm soil depth were continuously monitored throughout the experiment. A similar drought simulation was conducted at the same plots in the previous year.

### Labelling procedure

Pulse labelling with ^13^CO_2_ was conducted after at least 6 wk of drought simulation on three individual days with high radiation to ensure high uptake of labelled CO_2_ into plants (Supporting Information Table S1). Per labelling day, a set of drought and control plots were equipped with plexiglass chambers (1 × 1 × 0.7 m; 95% light permeability), which were placed on previously installed plastic frames (see Bahn *et al*., 2009, 2013). After 10 min of equilibration, ^13^CO_2_ (> 99.9% CO_2_ with 99 atom-% ^13^C; Cambridge Isotope Laboratories, Andover, MA, USA) was added over a period of 90 min, which has been shown to ensure sufficient ^13^C uptake for detecting ^13^C in microbial PLFAs (Bahn *et al*., 2013).

### Sample collection

Samples of plant biomass (aboveground biomass and fine roots) and soil were taken 1, 6, 7 and 8 wk (which was after mowing) after installation of rain-out shelters, as well as 1 wk after rewetting. For the pulse-labelling experiment, we took plant and soil samples 1 h before labelling to determine the natural background δ^13^C of plant biomass and soil (Table S2). We started sampling directly after the labelling procedure (at 0, 2 and 6 h), and continued on the following days (at 24, 48, 96 and 192 h). Then the study site was mown and aboveground biomass harvested. We took soil samples after mowing, and subsequently all plots were rewetted and sampling was continued (24, 72 and 192 h after rewetting). For each plant and soil sample, we pooled material from two soil collars with an area of 5 × 7 cm and a depth of 10 cm. The aboveground biomass was cut, and immediately treated by microwave for 3 min to interrupt any metabolic activities in plants (Popp *et al*., 1996). Soil was carefully sieved to 2 mm, manually freed from roots, which were collected (fine-root biomass), washed and also treated by microwave. Aliquots of soil samples for extraction of PLFAs were immediately frozen at −80°C, and remaining soil was stored at 4°C until further processing. Plant material and soil aliquots were dried (for 72 h at 60°C) and finely ground for subsequent analyses of bulk ^13^C content by EA-IRMS (elemental analysis-isotope ratio mass spectrometry; EA 1110 (CE Instruments, Milan, Italy), coupled to a Finnigan MAT Delta Plus IRMS (Thermo Fisher Scientific, Waltham, MA, USA)).

### Determination of C and N pools in the soil

Extractable organic carbon (EOC) and total extractable nitrogen (TEN) were measured in K_2_SO_4_ extracts (2 g of fresh soil was extracted with 20 ml of 0.5M K_2_SO_4_) using a Total Organic Carbon/Total Nitrogen (TOC/TN) analyser (TOC-V CPH E200V/TNM-122V; Shimadzu, Vienna, Austria). Aliquots of K_2_SO_4_ extracts were used to determine the δ^13^C of EOC, measured by direct injection (without column, direct mode) on high-performance liquid chromatography-isotope ratio mass spectrometry (HPLC-IRMS) (Dionex Corporation, Sunnyvale, CA, USA) linked to a Finnigan Delta V Advantage Mass Spectrometer connected by a Finnigan LC-IsoLink Interface (both Thermo Fisher Scientific, Waltham, MA, USA) at a flow of 0.5 ml water min^−1^. NH_4_^+^ was determined photometrically by a modified indophenol reaction method (Kandeler & Gerber, 1988) in K_2_SO_4_ extracts. NO_3_^−^ was determined in water extracts (2 g of fresh soil was extracted with 20 ml of water) by chemically suppressed ion chromatography (DX500; Dionex, Vienna, Austria) on a Dionex AS11 column.

### Soil microbial biomass

Soil microbial biomass was estimated using PLFAs, which were extracted from frozen soil samples as described in Kaiser *et al*. (2010). Briefly, total lipids were extracted from soil with chloroform/methanol/citric acid buffer and cleaned from neutral lipids using silica columns (LC-Si SPE; Supleco, Bellefonte, PA, USA) and chloroform, acetone and methanol. After addition of an internal standard (19:0), PLFAs were converted to fatty acid methyl esters (FAMEs) by alkaline methanloysis. Samples were analysed by gas chromatography using Trace GC Ultra connected with a GC-IsoLink to a Delta V Advantage Mass Spectrometer (both Thermo Fisher Scientific). Samples were injected in splitless mode (injector temperature: 230°C) and separated using a DB23 column (60 m × 0.25 mm × 0.25 μm; Agilent, Vienna, Austria) with 1.5 ml min^−1^ He as the carrier gas (GC programme: 1.5 min at 70°C, 30°C min^−1^ to 150°C, 1 min at 150°C, 4°C min^−1^ to 230°C, 15 min at 230°C). To identify single FAMEs, bacterial and fungal FAME mixtures (bacterial acid methyl ester mix and 37 Comp. FAME Mix; Supelco) were used; FAMEs were quantified against the internal standard (19:0). The concentrations and δ^13^C values of the identified FAMEs were corrected for the methyl group that was added during methylation. The sum of all subsequently described PLFAs was used as a proxy for total microbial biomass. We used the sum of i15:0, a15:0, i16:0, a16:0 and a17:0 for Gram-positive bacteria, 16:1ω7, 18:1ω7, cy17:0(9/10) and cy19:0(9/10) for Gram-negative bacteria and 15:0, 17:1ω6, 17:0, 18:1ωs5 and 10Me18:0 as general bacterial markers. Gram-positive, Gram-negative and general bacterial markers were summed to give total bacteria (Bacteria_tot_). We separated total fungal PLFAs (Fungi_tot_) into a general fungal marker (Fungi_gen_; 18:2ω6,9, 18:1ω9 and 18:3ω3,6,9) and 16:1ω5. The latter is often used as a marker for arbuscular mycorrhizal fungi, but also a marker for Gram-negative bacteria, especially in ecosystems with high bacterial abundance (Zelles, 1997; Olsson, 2006).

### Calculation of ^13^C excess

The excess ^13^C (in mg ^13^C m^−2^), which was the total amount of ^13^C in the individual C pools (per 1 m^−2^), was calculated as:


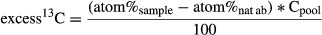


with atom%_sample_ being the atom% of the labelled sample, atom%_nat ab_ being the atom% of samples taken before labelling, therefore reflecting the natural ^13^C abundance of the samples (Table S2), and C_pool_ being the respective C pool (aboveground plant biomass and fine roots, bulk soil, EOC and soil microbial biomass, in mg C m^−2^). Note that C pools for soil, EOC and PLFAs, as well as for belowground biomass, were calculated only for the uppermost 10 cm of soil and were corrected for the respective soil bulk density (0.758 ± 0.03 and 0.722 ± 0.05 g cm^−3^ for drought and control plots, respectively; mean ± SE; *n *= 5).

### Statistical analysis

For single sampling points, differences between drought plots and controls for plant biomass C pools, soil and microbial groups were evaluated using one-way ANOVA. Effects of drought treatment and time (weeks of drought for C and N pools) or time after labelling (hours after labelling/rewetting for ^13^C excess data) were assessed using two-way repeated-measures ANOVA (level of significance: *P *<* *0.05). All data were tested for normality using the Shapiro–Wilk test, and for homoscedasticity using Levene’s test. If data did not meet the assumptions of ANOVA, they were log-transformed.

We conducted a canonical correspondence analysis (CCA; Anderson & Willis, 2003) to investigate the effects of abiotic parameters, including SWC and *T*_air_, as well as soil nutrient pools (EOC, TEN, NH_4_^+^ and NO_3_^−^ pools) and time (day of the year (DOY) and days of drought treatment (DOD)), on microbial community composition (using the relative abundance of all PLFAs) during the drought experiment (weeks 1, 6, 7 and 8 of drought and 1 wk after rewetting). The contribution of constrained variability to total community variability was used as a measure of the influence of environmental parameters on community variation. All statistical analyses were performed in R 2.15.2, using the vegan package for CCA (Oksanen *et al*., 2012).

## Results

### Microclimatic conditions and soil water content

During the experiment (10 June to 20 August), mean daily air temperature (*T*_air_) was 12.9°C, with a range from 1.8°C to 27.4°C (Fig. 1a). Mean daily soil temperature (*T*_soil_) at 10 cm soil depth was similar in drought plots (12.8°C) compared with controls (11.9°C) but showed greater daily amplitudes (day versus night temperatures) in drought plots (Fig. 1b). The total amount of precipitation excluded during 8 wk of drought simulation summed up to 363 mm, equalling to one-third of mean annual precipitation. SWC in drought plots was already slightly lower before the drought simulation (Fig. 1c), possibly resulting from exposure to drought in the previous year. However, the drought simulation caused a gradual decrease of SWC from 48.4 to 14.1% at 5 cm soil depth, the main rooting horizon, while in controls SWC varied between 53.3 and 38.8% during the treatment period. After rewetting, SWC steeply increased in the drought plots, but was still lower than in controls (Fig. 1c).

### Effects of drought on C pools in plants and soil

In the studied meadow, the mean (± SE; *n *=* *3) total aboveground biomass was 201 (± 16) g m^−2^ and the mean fine-root biomass was 358 (± 29) g m^−2^, but neither was significantly affected by the drought treatment, where mean aboveground biomass was 168 (± 35) g m^−2^ and mean root biomass was 381 (± 64) g m^−2^. Similar to biomass, neither the C content of aboveground biomass nor that of fine roots was altered by drought (Fig. S1a,d, Table 1a). Among all studied soil parameters, only NO_3_^−^ was significantly decreased during drought, by 55.4%, but this effect was not detectable after mowing (Fig. S1, Table 1a,b).

**Table 1:** Effects of drought, sampling time and the interaction term (drought × time) on carbon (C) and nitrogen (N) pools (a) before mowing, also given as percentage of controls, calculated from mean values for data obtained during drought treatment until mowing, and (b) after mowing, evaluated by two-way repeated-measures ANOVA

	(a) Before mowing							(b) After mowing					
	Drought			Time		Drought × time		Drought		Time		Drought × time	
	(%)	*F*(1)	*P*	*F*(2)	*P*	*F* (1, 2)	*P*	*F* (1)	*P*	*F* (1)	*P*	*F* (1,1)	*P*
Aboveground biomass	−17.4	1.96	ns	0.01	ns	0.01	ns						
Fine roots	+ 43.8	2.99	ns	0.05	ns	0.32	ns	1.34	ns	0.00	ns	0.02	ns
Soil	−0.8	0.03	ns	0.83	ns	0.20	ns	0.44	ns	1.91	ns	1.28	ns
EOC	+ 26.9	2.35	ns	1.76	ns	0.79	ns	4.02	ns	0.87	ns	2.41	ns
TEN	+ 22.4	1.31	ns	2.26	ns	1.25	ns	1.30	ns	1.52	ns	0.00	ns
NH_4_^+^	+ 20.9	1.41	ns	12.77	^*^^*^	2.60	ns	5.20	ns	1.45	ns	0.06	ns
NO_3_^−^	−**55.4**	4.70	^*^	1.24	ns	0.14	ns	2.64	ns	0.04	ns	0.11	ns
Total PLFAs	**+ 116.2**	33.57	^*^^*^^*^	1.33	ns	0.97	ns	1.27	ns	0.02	ns	0.46	ns
Fungi_tot_	**+ 107.3**	37.28	^*^^*^^*^	0.72	ns	1.10	ns	0.36	ns	0.26	ns	0.12	ns
Fungi_gen_	**+ 102.1**	37.79	^*^^*^^*^	0.78	ns	0.79	ns	0.01	ns	0.42	ns	0.01	ns
16:1ω5	**+ 117.2**	28.82	^*^^*^^*^	2.66	ns	1.37	ns	1.54	ns	0.08	ns	0.34	ns
Bacteria_tot_	**+ 121.5**	31.20	^*^^*^^*^	1.39	ns	0.80	ns	1.40	ns	0.01	ns	0.56	ns
Gram-negative	**+ 100.7**	29.39	^*^^*^^*^	1.17	ns	0.72	ns	2.54	ns	0.00	ns	0.57	ns
Gram-positive	**+ 181.7**	30.21	^*^^*^^*^	1.86	ns	1.24	ns	0.48	ns	0.00	ns	0.42	ns
Fungi:bacteria	−9.1	2.20	ns	3.68	ns	0.78	ns	1.06	ns	1.60	ns	1.67	ns

Before mowing, *n *=* *9 (for fine roots, *n *=* *6); after mowing, *n *=* *6. After mowing, no data for aboveground biomass were available. Values in bold show significant effects of drought calculated by one-way ANOVA. Asterisks indicate levels of significance (ns, not significant; ^*^, *P *<* *0.05; ^*^^*^, *P *<* *0.01; ^*^^*^^*^, *P *<* *0.001).

Bacteria_tot_, total bacterial PLFAs; EOC, extractable organic carbon; Fungi_gen_, general fungal PLFAs; Fungi_tot_, total fungal PLFAs; PLFA, phospholipid fatty acid; TEN, total extractable nitrogen.

### Effects of drought on soil microbes

**Figure 2:**
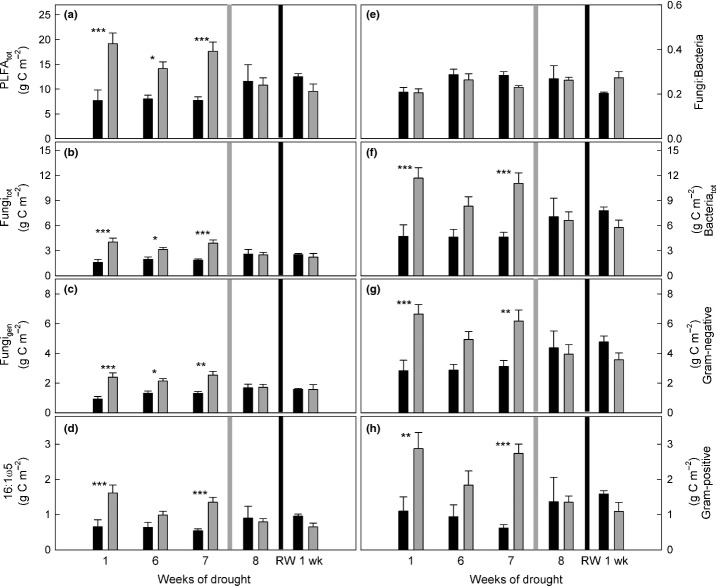
Microbial biomass over the course of the experiment (in g PLFA-C m^−2^ calculated for the uppermost 10 cm of soil; *n *=* *3; error bars show +SE) in control (black bars) and drought (grey bars) plots. (a) Total phospholipid fatty acids (PLFA_tot_) were divided into (b) total fungal (Fungi_tot_) and (f) total bacterial (Bacteria_tot_) PLFAs. Fungal PLFAs were further separated into (c) general fungal PLFAs (Fungi_gen_) and (d) 16:1ω5. Bacterial PLFAs were subdivided into (g) Gram-negative and (h) Gram-positive markers. (e) The ratio of Fungi_tot_ to Bacteria_tot_. Light grey lines indicate mowing of aboveground biomass, and black lines rewetting. Asterisks indicate significant differences between treatments at single samplings (one-way ANOVA; *, *P *<* *0.05; **, *P *<* *0.01; ***, *P *<* *0.001).

In drought-treated plots, total PLFAs were already significantly higher (+116.2%) compared with controls 1 wk after the beginning of the experiment and stayed at a constant higher level until mowing (Fig. 2a, Table 1a). This was similar for all microbial groups, but most pronounced for Gram-positive bacteria, which increased almost threefold compared with controls (+181.7%; Table 1a). The amounts of Gram-negative bacteria and total fungi were two-fold higher in drought plots than in controls (Fig. 2g, Table 1a); within fungal PLFAs, the marker 16:1ω5 showed the highest increases (Fig. 2d, Table 1a). Nevertheless, the ratio of fungal:bacterial PLFAs was not affected by the drought treatment (Fig. 2e, Table 1a). Interestingly, we found a strong decrease of total PLFAs by 37% (*t*-test; *P *=* *0.045) after mowing in the drought plots, while PLFAs in control plots were not affected (Fig. 2). Rewetting affected PLFAs neither in drought nor in control plots (Fig. 2, Table 1b).

**Figure 3:**
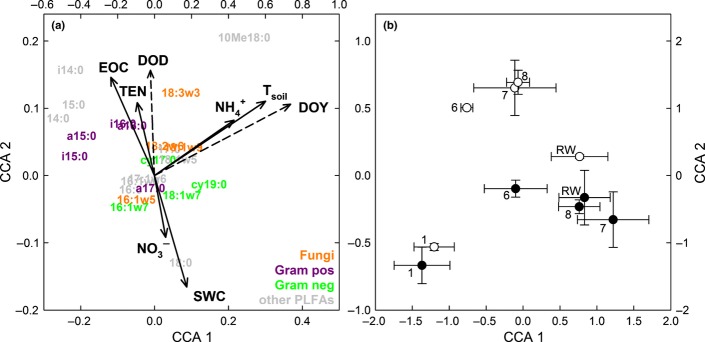
The effects of soil parameters and time on soil microbial community composition, as determined by canonical correspondence analysis (CCA). Relative abundances of phospholipid fatty acids (PLFAs) were used as the soil microbial community matrix, and soil parameters (gravimetric soil water content (SWC); soil temperature at 5 cm (*T*_soil_); extractable organic carbon (EOC); total extractable nitrogen (TEN); ammonium-N (NH_4_^+^); nitrate-N (NO_3_^−^)) and time (day of the year (DOY); days of drought (DOD) (dashed arrows)) were used as the environmental data matrix. Single PLFAs are coloured according to their attribution to microbial groups. The CCA is based on data from samples collected during drought (1, 6, 7 and 8 (after mowing) weeks of drought), as well as 1 wk after rewetting (RW). The contribution of constrained variability to total variability was 35%, of which CCA1 accounted for 60% and CCA2 for 15%. The significances of CCA1 and CCA2 were *P *=* *0.005 and *P *=* *0.15, respectively (permutation test). (a) Biplot of the distribution of single PLFAs and environmental parameters. (b) The distribution of samples collected during drought and after rewetting in control (closed circles) and drought (open circles) plots; characters next to symbols identify the time of sampling (error bars* *indicate SE).

We conducted a CCA to assess the effects of drought on the microbial community composition (Fig. 3). We used the relative abundances of all sampled PLFAs as the community matrix and DOY, DOD, SWC, *T*_soil_, and EOC, TEN, NH_4_^+^ and NO_3_^−^ pools as the environmental matrix. The contribution of the environmental variables (constrained variability) to the total variability of the microbial community matrix was 35%, of which CCA axis 1 (CCA1) accounted for 60% (*P *=* *0.005) and CCA axis 2 (CCA2) for 15% (*P *=* *0.13); thus, CCA1 accounted for 21% and CCA2 for 5% of total variability. We found that CCA1 showed a high correlation with DOY, *T*_soil_ and NH_4_^+^ (Fig. 3a); therefore, CCA1 could describe a seasonal gradient during the experiment, reflected by a distribution of the samplings along CCA1 (weeks 1–8 of drought, RW, 1 wk after rewetting; Fig. 3b). CCA2 displayed a gradient of SWC and showed a high correlation with DOD (Fig. 3a,b), showing the effects of the drought treatment on the microbial community composition. The drought-induced shift (CCA2) of the soil microbial composition was mainly driven by the distribution of Gram-positive and Gram-negative bacterial PLFA markers. Gram-positive bacteria correlated with higher EOC and TEN, and lower SWC, while Gram-negative bacteria correlated with higher SWC and NO_3_^−^. Fungal markers, by contrast, were not separated by SWC, but by NH_4_^+^ and *T*_soil_ (Fig. 3a).

### Effects of drought on C dynamics of plant, soil and microbial C pools

**Figure 4:**
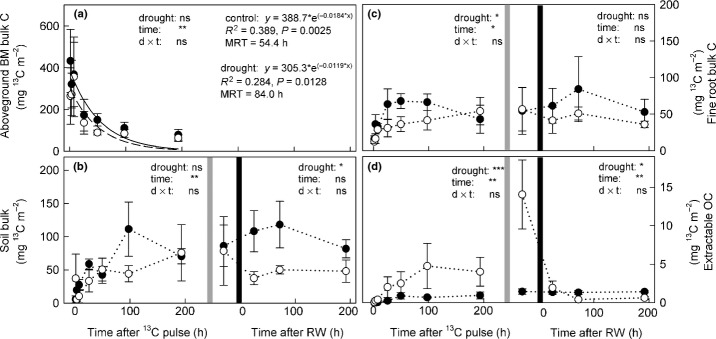
^13^C excess (in mg ^13^C m^−2^) after pulse-labelling in (a) aboveground plant biomass, (c) fine-root bulk biomass, (b) bulk soil and (d) the extractable organic carbon (C) pool in control (closed circles) and drought (open circles) plots; ^13^C excess in (a) followed an exponential decay function (*y *= *a* × *e*^(−λ*x)^; with *a* = initial pool size, λ = decay constant and *x* = time; control, solid line; drought, dashed line; mean residence time of ^13^C (MRT) = 1/λ). ^13^C excesses for (b) and (d) were calculated for the uppermost 10 cm of soil. Light grey lines indicate mowing, and black lines indicate rewetting. Effects of drought on ^13^C excess were analysed separately before and after cutting by two-way repeated-measures ANOVA with time and drought treatment as factors (ns, not significant; *, *P *<* *0.05; **, *P *<* *0.01; ***, *P *<* *0.001; for further details see Table 2).

In both controls and drought-treated plots, ^13^C in aboveground biomass peaked within 24 h after labelling (432.1 ± 151.0 and 263.7 ± 135.2 mg ^13^C m^−^², respectively; mean ± SE, *n* = 3) and then decreased exponentially over time (Fig. 4a). There was a trend towards higher mean residence time of ^13^C in aboveground biomass in drought plots, but differences compared with controls were not significant (Fig. 4a, Table 2a). Because mowing has to be considered as a major disturbance of C dynamics, ^13^C turnover was analysed separately before and after mowing by two-way repeated-measures ANOVA (Fig. 4b–d). During drought, before mowing, fine roots received significantly less ^13^C, while after mowing there was no difference compared with controls (Fig. 4c, Table 2a). Before mowing, ^13^C increased to the same extent in bulk soil of control and drought plots. After mowing and rewetting, however, the amount of ^13^C in the bulk soil increased further in the controls and became significantly higher than in drought plots (Fig. 4b). In contrast to the controls, where the amount of ^13^C in EOC increased only slightly, there was a significant accumulation of ^13^C in EOC in drought plots. Moreover, we detected a pulse of ^13^C in EOC directly after mowing in drought plots, which decreased after rewetting, while the amount of ^13^C in the controls constantly stayed at a low level (Fig. 4d).

**Table 2:** Effects of drought on ^13^C excess in all carbon (C) pools (a) before and (b) after mowing were analysed separately by two-way repeated-measures ANOVA

	(a) Before mowing						(b) After mowing					
	Drought		Time		Drought × time		Drought		Time		Drought × time	
	*F*(1)	*P*	*F*(6)	*P*	*F*(1, 5)	*P*	*F*(1)	*P*	*F*(3)	*P*	*F*(1,1)	*P*
Aboveground biomass	1.53	ns	4.89	^*^^*^	0.23	ns						
Fine roots	4.97	^*^	2.95	^*^	0.86	ns	0.75	ns	0.37	ns	0.24	ns
Soil	1.72	ns	3.81	^*^^*^	1.38	ns	8.71	^*^	0.23	ns	0.52	ns
EOC	7.93	^*^^*^	2.35	ns	1.05	ns	6.85	^*^	9.08	^*^^*^	8.89	^*^^*^
Total PLFAs	11.39	^*^^*^	5.73	^*^^*^^*^	0.64	ns	17.26	^*^^*^	0.13	ns	0.52	ns
Fungi_tot_	3.631	ns	6.18	^*^^*^^*^	0.27	ns	16.44	^*^^*^	0.54	ns	0.44	ns
Fungi_gen_	1.52	ns	5.53	^*^^*^^*^	0.10	ns	14.43	^*^^*^	0.85	ns	0.45	ns
16:1ω5	17.32	^*^^*^^*^	5.90	^*^^*^^*^	2.17	ns	21.52	^*^^*^^*^	0.10	ns	0.50	ns
Bacteria_tot_	14.67	^*^^*^^*^	4.46	^*^^*^	0.91	ns	17.39	^*^^*^	0.08	ns	0.71	ns
Gram-negative	30.81	^*^^*^^*^	8.40	^*^^*^^*^	2.04	ns	17.05	^*^^*^	0.14	ns	0.70	ns
Gram-positive	5.37	^*^	2.46	ns	1.51	ns	17.82	^*^^*^^*^	0.20	ns	0.88	ns
Fungi:bacteria	0.48	ns	3.32	^*^	1.51	ns	0.31	ns	5.11	^*^	1.12	ns

Before mowing, *n *=* *42; after mowing, *n *=* *12. After mowing, no data for aboveground biomass were available. Asterisks indicate levels of significance (ns, not significant; ^*^, *P *<* *0.05; ^*^^*^, *P *<* *0.01; ^*^^*^^*^, *P *<* *0.001).

Bacteria_tot_, total bacterial PLFAs; EOC, extractable organic carbon; Fungi_gen_, general fungal PLFAs; Fungi_tot_, total fungal PLFAs; PLFA, phospholipid fatty acid.

**Figure 5:**
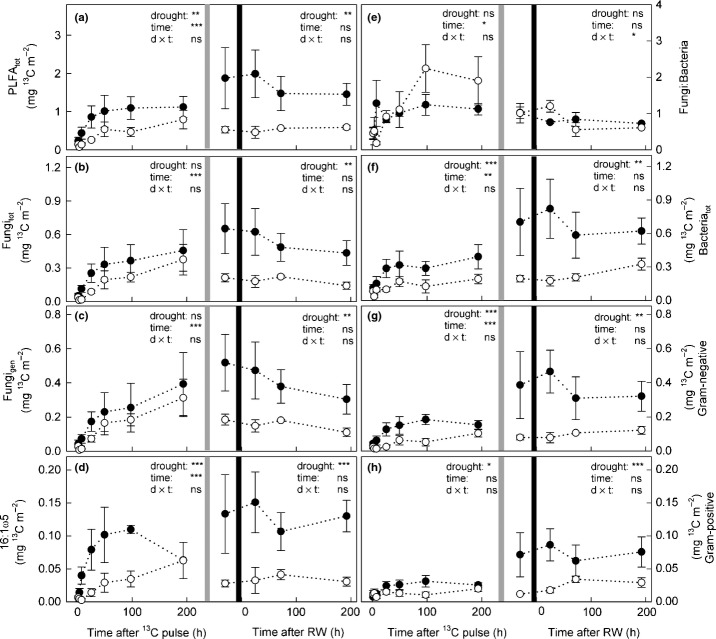
^13^C excess (in mg ^13^C m^−2^) after pulse-labelling in (a) total phospholipid fatty acids (PLFA_tot_), and (b) total fungal (Fungi_tot_) and (f) total bacterial (Bacteria_tot_) PLFAs, and (e) the ratio of fungal to bacterial ^13^C uptake in control (closed circles) and drought (open circles) plots over time. Fungal PLFAs were grouped into (c) general fungal marker and (d) 16:1ω5 PLFAs, and bacterial PLFAs into (g) Gram-negative and (h) Gram-positive PLFAs. Values were calculated for the uppermost 10 cm of soil. Mowing is indicated by light grey lines and rewetting is indicated by black lines. Effects of drought before and after cutting were analysed separately by two-way repeated-measures-ANOVA (ns, not significant; *, *P *<* *0.05; **, *P *<* *0.01; ***, *P *<* *0.001; further details are given in Table 2).

Although microbial biomass was higher in drought plots (Fig. 2), the absolute amount of ^13^C incorporated into total PLFAs was significantly lower in drought plots compared with controls (Fig. 5a). This reduction during drought was most pronounced for Gram-negative bacteria, followed by the fungal marker 16:1ω5 and Gram-positive bacteria (Fig. 5d,g,h). For the general fungal marker, in contrast, similar amounts of ^13^C were incorporated in drought plots as in the controls (Fig. 5c). In both control and drought plots, we found a remarkably high ratio of fungal:bacterial ^13^C uptake (Fig. 5e), in contrast to the lower fungi:bacteria PLFA ratio (Fig. 2e), indicating that fungi received a higher portion of plant-derived C compared with bacteria.

After mowing, ^13^C in PLFAs in drought-treated plots stayed at a significantly lower level than in controls (Fig. 5a). This pattern was similar for all microbial groups, and also for fungal markers, where ^13^C incorporation had not been affected by drought before mowing (Fig. 5b,c). This indicated that mowing caused a pulse of plant C in the soil, which in control plots was incorporated into PLFAs, but in drought plots was not taken up by microbes and accumulated in the EOC pool. However, mowing caused a significant decrease in microbial biomass in drought-treated plots and therefore dead microbes may also have substantially contributed to this label peak in the EOC (Figs 2a, 4d).

## Discussion

The probability of extreme events and drought periods is predicted to increase in European mountain regions (IPCC, 2012), with potentially severe consequences for ecosystem C dynamics (Reichstein *et al*., 2013). However, the effects of drought on ecosystems that are usually well supplied with water are not well understood. In this study, we demonstrate that under field conditions a severe summer drought period has the potential to weaken the coupling of plant and microbial C turnover in a mountain meadow.

### Effects of drought on plant belowground C allocation

Drought is well known to strongly affect plant biomass and C pools, by increasing the proportion of fine-root biomass to sustain water uptake and photosynthesis, at the expense of aboveground C stocks (Chaves *et al*., 2003; McDowell, 2011). However, in our experiment, drought neither affected plant biomass, nor their C pools, probably because most of the plant biomass was already largely established at the onset of drought. Nonetheless, drought significantly reduced the absolute amount of recently assimilated C in fine roots, without decreasing C uptake at the time of pulse labelling (Fig. 4c). Thus, drought may have slowed down plant C turnover, which was also found by others (Ruehr *et al*., 2009; Barthel *et al*., 2011; Brüggemann *et al*., 2011). However, ^13^C significantly increased in the EOC pool during drought, which suggests that plant roots may have maintained, or even increased root exudation or the release of mucilaginous material to decrease the friction resistance in the soil and keep the contact between roots and soil (Walker *et al*., 2003).

### Effects of drought on soil microbes

In contrast to our expectations, both fungal and bacterial PLFAs were higher in drought-treated plots than in controls. In addition, we detected a shift of the microbial community composition that occurred mainly within soil bacteria. We also observed that drought showed a distinct effect on the incorporation of plant-derived C into microbial PLFAs, specifically a reduction of label in bacteria. Fungal PLFAs were increased during drought and incorporated similar amounts of recent plant-derived C in drought plots as in controls (Fig. 5b). This corresponds well with the finding of Bahn *et al*. (2013) at the same study site, where even extended reductions in photosynthesis did not decrease the amount of recent plant C in fungal PLFAs. Thus, the transfer of ^13^C label from plants to fungi seemed not to be interrupted by experimental drought. Of the four PLFAs used as fungal markers, only 16:1ω5, often used as a marker for arbuscular mycorrhizas, showed a significant reduction in ^13^C incorporation during drought. This was in contrast to our expectations, as mycorrhizal fungi are a strong sink for plant-derived C in meadows (Denef *et al*., 2009; Balasooriya *et al*., 2012) and may even promote water uptake for plants during drought conditions (Wardle *et al*., 2004; Allen, 2007). However, this biomarker might not solely be representative of arbuscular mycorrhiza, as it was shown also to occur in bacterial taxa (Olsson, 2006).

All bacterial PLFAs were increased in experimental drought plots, but Gram-positive bacteria became relatively more abundant compared with Gram-negative bacteria. Gram-positive bacteria are considered to be inherently more resistant to drought regarding their cell wall composition (Schimel *et al*., 2007; Manzoni *et al*., 2012a), and their abundance correlated with higher EOC and TEN (Fig. 3a). This is further evidence that, in contrast to Gram-negative bacteria, Gram-positive bacteria may prefer more complex substrates and therefore could be classified as ‘dry-adapted generalists’ (Lennon *et al*., 2012). Many Gram-negative bacteria in turn seem to benefit from root or fungal exudates (Treonis *et al*., 2004; Olsson, 2006; Denef *et al*., 2009; Balasooriya *et al*., 2012; Bahn *et al*., 2013) and incorporated almost tenfold more recent plant C than Gram-positive bacteria, but both received significantly less ^13^C during drought.

Thus, drought weakened the link between both Gram-positive and Gram-negative bacteria and plants, as indicated by a significantly lower absolute bacterial uptake of label and a concurrent increase of ^13^C in the EOC. This could have been caused by decreased bacterial activity and a higher proportion of dormant microbes (Schimel *et al*., 2007) and consequently lower uptake of plant-derived C during drought, which is, however, in contrast to the observed strong increase in bacterial PLFAs. It is more likely that drought may not have caused a decrease in microbial activity, which was also reported by Steinweg *et al*. (2013), but restricted it to water-containing, but disconnected, soil pores (Schimel *et al*., 2007; Moyano *et al*., 2013). These small soil pores may represent ‘hot spots’ for bacterial decomposers, and may not be accessible to soil mesofauna grazing on microbes, or competing plant roots or fungal hyphae (Strickland & Rousk, 2010). Microbes in these disconnected microsites may be separated from root exudates, which would explain the reduced microbial uptake of plant-derived ^13^C from EOC.

We rejected our hypothesis that drought decreases microbial biomass and showed that, in the studied grassland, beneficial effects on microbes may have exceeded detrimental ones, possibly also reflecting a carry-over effect from the drought experiment performed in the same plots in the previous year (but see results on recovery responses after rewetting). This is in contrast to other studies in Mediterranean or semi-arid grasslands, where drought induced a decrease in microbial biomass (Alster *et al*., 2013) or diversity, especially for bacteria (Yuste *et al*., 2011; Sheik *et al*., 2011). Microbial biomass also decreased when a drought treatment was applied to barren soil, but increased in soils with mixed plant vegetation (Sanaullah *et al*., 2011). This illustrates that effects of drought are strongly context-dependent, but also suggests that the presence of plants is crucial for microbial responses to drought.

### Mowing and rewetting

Mowing has been shown to lead to a short-term pulse of plant-released C into the rhizosphere (Paterson & Sim, 1999; Hamilton *et al*., 2008; Henry *et al*., 2008). While we expected that this mowing-induced C pulse would be reduced by drought, we observed an increase of ^13^C in both drought plots and controls. In controls, this pulse of C after mowing was visible as a significant increase of ^13^C in microbial PLFAs, whereas in drought plots ^13^C peaked in the EOC pool and not in the microbes. This further indicates that microbes may have been unable to take up the C released by plants during drought. Alternatively, the steep increase of ^13^C in EOC in drought plots may have been fuelled by lysis of dead microbes or by an increase in fine-root turnover after mowing. However, such C pulses after mowing have been shown to induce a short-term increase in nutrient mineralization, which in turn increases the availability of nutrients for plants (Hamilton *et al*., 2008). Thus, smaller C pulses might lead to lower nutrient mineralization rates and may increase the competition for nutrients between plants and microbes, and so hamper plant growth.

In contrast to mowing, rewetting did not affect microbial biomass either in controls or after drought treatment. The ^13^C peak in the EOC in drought plots after mowing quickly disappeared, but was not used for new biomass production, as evidenced by a lack of ^13^C incorporation into PLFAs. In turn, after rewetting, soil respiration rates steeply increased (Ladreiter *et al*., data not shown; ‘Birch-effect’ (Birch, 1958)), which suggests that the accumulated ^13^C might have become available for microbes after plots were rewetted, or it may have leached out of the system. Nonetheless, we observed a shift of the microbial community composition in the drought-treated plots towards that of the controls already 1 wk after rewetting, indicating that the microbial community in this mountain grassland is highly resilient, even to strong drought.

### Conclusions

Severe experimental drought, surprisingly, did not reduce microbial biomass, but altered the microbial community composition in a mountain meadow in favour of Gram-positive bacteria. Drought did not interrupt the C transfer from plants to fungi, but decreased the uptake of label into bacterial groups, especially when the meadow was mown. From our study, we therefore conclude that (1) drought has the potential to weaken the link between plant and bacterial C turnover and (2) the microbial community in the studied grassland is highly adaptable to altered soil moisture conditions, responding to severe drought by shifting to slow-growing, drought-adapted soil microbes, such as Gram-positive bacteria, and quickly recovers after rewetting.

## Supporting Information

**Fig S1** Plant biomass carbon pools, and soil carbon and nitrogen pools over the course of the drought experiment. **Table S1** Date and time, and abiotic conditions during pulselabelling, for individual sites **Table S2** Natural abundance of ^13^C of plant biomass, soil and extractable organic C pool in the soil
