# Supplementary material for: Experimental drought reduces the transfer of recently fixed plant carbon to soil microbes and alters the bacterial community composition in a mountain meadow
Source: New Phytol. 2013 Oct 31;201(3):916–27. doi: 10.1111/nph.12569 (PMC3908363; doi:10.1111/nph.12569)
Supplement: Supplementary file 1 — Fig S1 Plant biomass carbon pools, and soil carbon and nitrogen pools over the course of the drought experiment. Table S1 Date and time, and abiotic conditions during pulselabelling, for individual sites Table S2 Natural abundance of 13C of plant biomass, soil and extractable organic C pool in the soil [file nph0201-0916-sd1.docx]

**Supporting information Fig. S1, Tables S1 & S2**

**Fig. S1** Carbon pools in (a) aboveground and in (d) fine root bulk biomass, as well as pools of (b) extractable organic C (EOC) (e) total extractable N (TEN), (c) ammonium (NH4-N) and (f) nitrate (NO3-N) in soils of control (black bars) and drought plots (grey bars) over the time of the drought experiment. Bars display mean values (n=3, error bars = SE). Grey lines indicate mowing, black lines indicate rewetting. Asterisks display significant differences between treatments at single sampling points (derived by one-way ANOVA, *, *P* < 0.05; **, *P* < 0.01; *, *P* < 0.05).

**Table S1** *Date* and time of the day (*time*) of labelling events for drought and control plots respectively. *[CO_2_]_start_* and *[CO_2_]_labelling_* show the CO_2_-concentrations in the labelling chamber before and during labelling

| *Date* | *Treatment* | *Time*  *(MET)* | *[CO_2_]_start_*  *(ppm)* | *[CO_2_]_label_*  *(ppm)* | *^13^CO_2_*  *(atom%)* | *^13^CO_2 add_*  *(ml min^-1^)* | *PAR*  *(µmol m^-2^s^-1^)* | *T_in_*  *(°C)* | *T_out_*  *(°C)* |
| --- | --- | --- | --- | --- | --- | --- | --- | --- | --- |
| 22.07.2010 | *drought* | 11:54–13:24 | 237 | 552 ± 19 | 18.3 ± 1.1 | 12.9 | 1848–1908 | 26.7–37.7 | 26.0–27.3 |
|  | *control* | 12:49–14:19 | 248 | 556 ± 5 | 23.2 ± 0.1 | 38.3 | 1423–1908 | 29.7–39.9 | 26.5–28.8 |
| 28.07.2010 | *drought* | 10:40–12:10 | 253 | 519 ± 18 | 22.6 ± 0.2 | 23.3 | 652–1185 | 19.1–24.1 | 15.7–19.4 |
|  | *control* | 11:11–12:41 | 256 | 496 ± 28 | 18.9 ± 0.6 | 21.7 | 652–1185 | 15.0–25.8 | 14.9–21.2 |
| 31.07.2010 | *drought* | 12:05–13:35 | 280 | 520 ± 12 | 23.5 ± 0.3 | 34.2 | 1840–2026 | 23.4–33.2 | 21.1–24.2 |
|  | *control* | 12:49–14:19 | 216 | 555 ± 28 | 22.9 ± 0.5 | 43.2 | 1759–1993 | 24.9–30.1 | 20.5–24.4 |

*^13^CO_2_* describes the isotopic composition in atom% during labelling (values are means of 5 min intervals ± SE) analysed by Quantum cascade laser (QCL, Aerodyne Research Inc., Billerica, MA, USA), the average amount of *^13^CO_2_ added* is given in ml min^-1^. Photosynthetic active radiation (*PAR*) during labelling as well as air temperature inside (*T_in_*) and outside (*T_ou_*_t_) the chambers are displayed (minimum – maximum temperature). CO_2_ concentration in the labelling chamber was monitored with infrared-gas-analysers (Vaisala Ayj, Vantaa, Finland and EGM-4, PP Systems, Hitchin, UK).

**Table S2** Natural abundance ^13^C of several components sampled from drought and control plots

|  | *Control* | | |  | *Drought* | | |  |
| --- | --- | --- | --- | --- | --- | --- | --- | --- |
|  | *mean* |  | *SE* |  | *mean* |  | *SE* | *p* |
| Aboveground BM bulk | -28.12 | ± | 0.09 |  | -27.89 | ± | 0.25 | ns |
| Fine root bulk | -27.89 | ± | 0.16 |  | -26.89 | ± | 0.37 | ns |
| Soil bulk | -26.53 | ± | 0.08 |  | -26.40 | ± | 0.25 | ns |
| EOC | -26.84 | ± | 0.48 |  | -26.51 | ± | 0.15 | ns |

Values represent means (*n* = 3) ± SE. Differences between treatments were tested using *t*-tests, if data was not homoscedastic Welch-test was used instead. Isotopic composition of biomass samples are reported in δ^13^C (‰): $\delta{}^{13}{C_{sample}=1000* \frac{R_{sample-}R_{standard}}{R_{standard}}}$ with $R$ describing the ratio of ^13^C/^12^C. The δ^13^C values were calculated relative to the VPDB standard ($R_{standard}$).
